# Assessment of mycotoxins in cornflakes marketed in Lebanon

**DOI:** 10.1038/s41598-023-48172-8

**Published:** 2023-11-28

**Authors:** Hussein F. Hassan, Farah Awada, Hani Dimassi, Christina El Ahmadieh, Nour Bachar Hassan, Sami El Khatib, Nisreen Alwan, Mohamad G. Abiad, Mireille Serhan, Nada El Darra

**Affiliations:** 1 Department of Natural Sciences, School of Arts and Sciences, Lebanese American University, Beirut, Lebanon; 2 Faculty of Health Sciences, Beirut Arab University, Beirut, Lebanon; 3 School of Pharmacy, Lebanese American University, Byblos, Lebanon; 4 Department of Food Sciences and Technology, School of Arts and Sciences, Lebanese International University, Bekaa, Lebanon; 5 Department of Biological Sciences, School of Arts and Sciences, Lebanese International University, Bekaa, Lebanon; 6 Center for Applied Mathematics and Bioinformatics (CAMB) at Gulf University for Science and Technology, Hawally, Kuwait; 7 College of Health Sciences, Abu Dhabi University, Abu Dhabi, United Arab Emirates; 8 Department of Nutrition and Food Sciences, Faculty of Food and Agricultural Sciences, American University of Beirut, Beirut, Lebanon; 9 Department of Nutritional Sciences, Faculty of Health Sciences, University of Balamand, Deir el Balamand, Tripoli, Lebanon

**Keywords:** Medical research, Chemistry

## Abstract

Cornflakes are a popular and convenient breakfast cereal made from corn and widely consumed worldwide, including in Lebanon. However, they are susceptible to mycotoxin contamination, which can have harmful effects on human health. Our study evaluated the occurrence of five mycotoxins (AFB1, OTA, FUM, ZEA, DON) levels in packed cornflakes marketed in Lebanon. A market screening identified 35 different cornflake stock-keeping units (SKU) in the Lebanese market, originating from 10 different brands and having different tastes and shapes. SKUs were collected and tested for five mycotoxins in triplicates using enzyme-linked immunosorbent assay technique. The results showed the presence of the five mycotoxins in the samples. The average levels of AFB1, OTA, ZEA and FUM among positive samples (above limit of detection) were 1.58, 1.2, 15.1 and 774.1 μg/kg, respectively, and were below the EU limits. On the other hand, the average level of DON was 1206.7 μg/kg, exceeding the EU limit. Furthermore, out of the positive samples, 60%, 17%, 9%, 14%, and 6% exceeded the EU limits for DON, OTA, AFB1, FUM, and ZEA, respectively. Notably, SKUs made in Lebanon had significantly (*p* < 0.05) higher levels of AFB1 and FUM. The packing size of the cornflakes had no significant (*p* > 0.05) effect on the levels of the five mycotoxins detected in the samples. AFB1, FUM and ZEA levels differed significantly among SKUs (*p* > 0.05). Considering these findings, further studies should be conducted to assess the exposure to mycotoxins from the consumption of cornflakes in Lebanon, especially among children.

## Introduction

Mycotoxins are naturally occurring toxic compounds with high stability under thermal conditions and a remarkable ability to accumulate in living organisms^1^. The term “Mycotoxin” refers to low molecular weight compounds (0.3–0.7 kDa) that are produced as secondary metabolites by certain significant fungal genera, including *Fusarium*, *Penicillium*, *Aspergillus*, and *Alternaria*, during pre- and post-harvest storage^2^. Among the more than 300 identified secondary compounds, deoxynivalenol (DON), ochratoxin A (OTA), zearalenone (ZEN), and aflatoxins (AFs) have been the most extensively studied mycotoxins. However, other mycotoxins such as patulin, fumonisin B1 (FB1), fumonisin B2 (FB2), and citrinin have also garnered significant attention in research investigation. A variety of cereals, dried fruits, nuts, cocoa, spices, and coffee are susceptible to mycotoxin contamination, primarily involving OTA, ZEN, DON, and AFs^3^. Many countries have implemented maximum acceptable levels for the presence of mycotoxins in consumed food, which requires sensitive and selective methods for their determination and quantification. High-performance liquid chromatography (HPLC) without or with a fluorescence detection (HPLC-FD), liquid chromatography (LC) coupled to mass spectrometry (MS) detector, thin-layer chromatography (TLC) and enzyme-linked immunosorbent assay (ELISA) are the analytical methods used to quantify mycotoxins in cereals^4^.

Research on mycotoxin contamination in maize crops, as well as maize-based food and feeds, has been extensively investigated and documented on a global scale. Given that mycotoxins are naturally-occurring harmful secondary metabolites, their presence in food is inevitable and challenging to predict. Therefore, ensuring food safety necessitates dedicated efforts to minimize their occurrence to the lowest feasible levels. Consequently, in order to assess the potential risks faced by consumers when consuming contaminated food, it becomes imperative to gather data regarding the concentrations and distribution of mycotoxins in food products intended for human consumption^5^.

Given that a significant proportion of the Lebanese population heavily relies on cereals, which make up approximately 35% of the daily energy intake for adults, there is an elevated vulnerability to mycotoxin exposure^6^. In Lebanon, several studies assessed mycotoxins in commodities, such as breast milk, infant formula, tea, thyme and dairy products, and rice^7–12^. Consequently, it becomes essential to prioritize the assessment of mycotoxin levels in the foods consumed in Lebanon to include cornflakes. Therefore, the aim of our study is to measure mycotoxins in cornflakes marketed in Lebanon.

## Materials and methods

### Sample collection and analysis

Lebanese market was screened for cornflake stock-keeping units (SKUs) in summer 2022, and 35 SKUs originating from 10 different brands and having different tastes and shapes, were identified. Determination of mycotoxins was carried out in triplicates using the Enzyme-Linked Immunosorbent Assay (ELISA) technique. For this, RIDASCREEN AFB1 30/15 (R1211), OTA 30/15 (R1312), ZEA (R1401), FUM (R3401) and DON (R5906) test kits (R-biopharm, Germany) were used. Test kit has 96 wells. Kit manuals were followed step by step. In brief, 5 g of ground and homogenized sample was mixed with 70% methanol and filtered. Then, obtained filtrate was diluted with distilled water, and used in the test wells. Test wells were added to the holder. Then, standard/samples and enzyme conjugate were added into wells. Plate was gently shaken, and then incubated for half an hour at room temperature in a dark place. After that, liquid was poured out of the wells and then washed with a washing solution. In addition, substrate/chromogen followed by stop solution were added to each well. Absorbance was determined at 450 nm by a plate spectrophotometer within 15 min of adding the stop solution. Mycotoxin concentration corresponding to the absorbance of each sample can be obtained from the calibration curve via the cubic spline function of the RIDA®SOFT Win (Art. No. Z9999) software. Detections limits indicated by the kit manufacturer for AFB1, OTA, ZEA, FUM and DON were 1 µg/kg, 0.5 µg/kg, 1750 ng/kg, 25 µg/kg and 18.5 µg/kg.

### Statistical analysis

Mycotoxins concentration was calculated as a mean of two replicates. Data were processed in SPSS V27. Proportion of sample with occurrence of mycotoxin was compared by different sample characteristics, where frequency and percentage were reported, and the Pearson’s chi-square was used to generate the *p* value. In instances where the count of expected cells was less than permitted the fisher exact test was utilized to generate the *p* values. All analyses were done at the 5% significance level.

## Results and discussion

Sample characteristics are presented in Table 1. Average, minimum and maximum concentrations of different mycotoxins, and effects of country of origin, pack size and brand on mycotoxins, are presented in Tables 2 and 3, respectively.

**Table 1:** Characteristics of the cornflake samples.

Characteristic	N = 35
Country of origin	
Lebanon	16
All others (Ukraine, France, Turkey, Poland, UK)	19
Pack size	
250 g and less	14
More than 250 g	21
Brand	
1	7
2	2
3	10
4	4
5	2
6	2
7	1
8	1
9	2
10	4

**Table 2:** Average and range of results for different mycotoxins.

	Average	Minimum	Maximum	EU limit	Number of samples (%) above EU limits
DON (μg/kg)	1206.7 ± 60.3	100.9	3750.0	500	21 (60%)
OTA (μg/kg)	1.2 ± 0.08	3.3	8.6	3	6 (17%)
AFB1 (μg/kg)	1.6 ± 0.09	1.1	2.9	2	3 (9%)
FUM (μg/kg)	774.1 ± 69.7	30.0	6285.0	1000	5 (14%)
ZEA (μg/kg)	15.1 ± 0.76	10.4	326.3	50	2 (6%)

**Table 3:** Effect of country of origin, pack size, and brand on mycotoxins in cornflakes.

Variable	Not detected		Detected		*p* value		Variable	Not detected		Detected		*p* value	
	N	%	N	%				N	%	N	%		
AFB1							OTA						
Lebanon versus others							Lebanon versus others						
Lebanon	5	31.3	11	68.8	0.014	Significant	Lebanon	13	81.3	3	18.8	0.999	Not significant
All others (Ukraine, France, Turkey, Poland, UK)	14	73.7	5	26.3			All others (Ukraine, France, Turkey, Poland, UK)	15	78.9	4	21.1		
Size							Size						
250 g and less	6	42.9	8	57.1	0.317	Not significant	250 g and less	10	71.4	4	28.6	0.401	Not significant
More than 250 g	13	61.9	8	38.1			More than 250 g	18	85.7	3	14.3		
Brand							Brand						
1	6	85.7	1	14.3	0.007	Significant	1	5	71.4	2	28.6	0.312	Not significant
2	2	100	0				2	2	100	0			
3	2	20	8	80			3	7	70	3	30		
4	3	75	1	25			4	4	100	0	0		
5	0	0	2	100			5	2	100	0	0		
6	2	100	0	0			6	2	100	0	0		
7	1	100	0	0			7	0	0	1	100		
8	0	0	1	100			8	0	0	1	100		
9	2	100	0	0			9	2	100	0	0		
10	1	25	3	75			10	4	100	0	0		
DON							FUM						
Lebanon versus others							Lebanon versus others						
Lebanon	1	6.3	15	93.8	0.347	Not significant	Lebanon	2	12.5	14	87.5	< .001	Significant
All others (Ukraine, France, Turkey, Poland, UK)	4	21.1	15	78.9			All others (Ukraine, France, Turkey, Poland, UK)	14	73.7	5	26.3		
Size							Size						
250 g and less	0	0	14	100	0.069	Not significant	250 g and less	5	35.7	9	64.3	0.332	Not significant
More than 250 g	5	23.8	16	76.2			More than 250 g	11	52.4	10	47.6		
Brand							Brand						
1	1	14.3	6	85.7	0.117	Not significant	1	6	85.7	1	14.3	< .001	Significant
2	2	100	0	0			2	1	50	1	50		
3	1	10	9	90			3	0	0	10	100		
4	0	0	4	100			4	3	75	1	25		
5	0	0	2	100			5	1	50	1	50		
6	1	50	1	50			6	2	100	0	0		
7	0	0	1	100			7	1	100	0	0		
8	0	0	1	100			8	0	0	1	100		
9	0	0	2	100			9	2	100	0	0		
10	0	0	4	100			10	0	0	4	100		
ZEA													
Lebanon vs others													
Lebanon	16	100	0	0	0.109	Not significant							
All others (Ukraine, France, Turkey, Poland, UK)	15	78.9	4	21.1									
Size													
250 g and less	12	85.7	2	14.3	0.999	Not significant							
More than 250 g	19	90.5	2	9.5									
Brand													
1	5	71.4	2	28.6	0.041	Significant							
2	0	0	2	100									
3	10	100	0	0									
4	4	100	0	0									
5	2	100	0	0									
6	2	100	0	0									
7	1	100	0	0									
8	1	100	0	0									
9	2	100	0	0									
10	4	100	0	0									

Our results show that all five mycotoxins were present in cornflakes marketed in Lebanon. In particular, among positive samples, 60% exceeded the EU limit for DON, 17% for OTA, 9% for AFB1, 14% for FUM, and 6% for ZEA (Table 2). Average levels of AFB1, OTA, FUM and ZEA in the cornflake samples were below the maximum permissible values, that of DON exceeded the maximum permissible value (Table 2). In other words, DON presents the highest concern toward its presence compared to other mycotoxins. Although AFB1 average (1.58 μg/kg) was below the EU limit of 2 μg/kg, and 3 (9%) samples only exceeded this limit, this should be of concern as AFB1 is classified as class 1 carcinogen, unlike the other 4 mycotoxins, by the International Association for Research on Cancer (IARC)^13^.

Our results found that the brand name had a significant (*p* < 0.5) effect for AFB1, FUM, and ZEA, and not for OTA and DON contamination levels. On the other hand, the country of origin (Lebanon vs. others) had a significant (*p* < 0.5) effect on AFB1 and FUM, and not on OTA, ZEA and DON. In contrast, pack size (less or above 250 g) had no significant effect on the levels of 5 mycotoxins (Table 3).

Given that no study was done in Lebanon on cornflakes, we will compare our results to a study done by Hassan et al. (2022) on another cereal, rice. Using ELISA, Hassan et al. (2022) assessed OTA in rice brands available in Lebanon and United Arab Emirates (UAE). OTA was found in 56 (53%) samples in Lebanon and 73 (58%) samples in the UAE. Only one sample (1%) in Lebanon had a level at the borderline of the European Union (EU) limit, and two samples (1.6%) in the UAE had a level above the EU limit (5 μg/kg). In our study, no significant difference was found between SKUs from Lebanon versus other countries in terms of OTA, which goes in line with the findings of^10^. In addition, average concentration of OTA in our study was 1.2 μg/kg, which is close similar to the one reported by^10^.

Cereals are commonly contaminated with various mycotoxins and fungal metabolites, with wheat and maize having the highest concentrations of FBs, DON, AFs, and ZEN among reported cereals^14^. The presence of DON has been repeatedly detected in cereal-based products collected from markets over the last years. The most important mycotoxins in corn worldwide are aflatoxins, fumonisins, trichothecenes (especially deoxynivalenol), and zearalenone. The most common secondary fungal metabolites were found to be Fusarium mycotoxins, with DON being the most occurring present in 73.7% of the samples^15^. This goes in line with our findings, where 60% of the samples had DON exceeding the EU limit.

A study conducted in Pakistan found that a significant proportion of breakfast cereals were contaminated with mycotoxins. Specifically, 41% of the samples were contaminated with AFs, and 16% of the positive samples exceeded the EU limit for AFB1 (vs. 9% in our study), and 8% exceeded the EU limit for total AFs. Additionally, 48% of the samples were contaminated with OTA, with 30% exceeding the EU limit (vs. 17% in our study), and 53% of the samples were contaminated with ZEA, with 8% exceeding the EU limit (vs. 6% in our study)^4^. In other words, the % of samples above limits were in general higher in the Pakistani study.

A metanalysis study was conducted one by^2^ to estimate the prevalence and concentration of total aflatoxin (TAF), OTA, ZEA and DON in bread, cornflakes, breakfast cereals and pasta-based products. The prevalence and concentration of studied mycotoxins varied with the studied cereal-based foods. In this context, the overall rank order of mycotoxins prevalence in the cereal foods was OTA > DON > ZEN > TAF > 15-ADON > 3-ADON. Furthermore, the overall rank order of mycotoxins based on concentration in the cereal foods investigated was DON > ZEN > 15-ADON > OTA > 3-ADON > TAF. This goes in line with our study which found that DON is the most prevalent mycotoxin. While the meta-analysis study provides a broader overview of the prevalence and concentration of mycotoxins in various cereal-based foods, our research focuses specifically on cornflakes marketed in Lebanon.

A study from Portugal evaluated the occurrence of twenty-one mycotoxins and metabolites in breakfast cereals specifically targeted towards children. The findings revealed that 96% of the samples tested positive for multiple mycotoxins, with 21 different combinations consisting of two to seven types of mycotoxins being identified^16^.

Over a 2-year period, a total of 489 samples of breakfast cereal samples made from corn, rice, wheat, and oat were gathered from retail market in the United States. Out of these, 205 samples (42%) were found to contain OTA, ranging from 0.10 to 9.30 ng/g with most levels falling below the EU limit, except in 16 samples of oat-based cereals. Oat-based breakfast cereals had the highest incidence of OTA (70%, 142/203), followed by wheat-based (32%, 38/117), corn-based (15%, 15/103), and rice-based breakfast cereals (15%, 10/66). Overall, the levels of mycotoxins varied between cereal types and brands, indicating that some cereals and brands may be more prone to contamination than others^17^. In Serbia, a study aimed to investigate the presence of OTA and AFB1 in breakfast cereals. A total of 136 samples were collected in 2012 and 2015 and analyzed using high-performance liquid chromatography with fluorescence detection. The results revealed that OTA was present in 20.7% of the samples in 2012 and 13.0% in 2015, with 3.6% and 0% of the samples exceeding EU maximum level, respectively. AFB1 was detected in low levels in 11.1% of the samples collected in 2015, primarily in corn-based cereals^18^. Another study in Serbia by^19,20^ reported the presence of ZEA in 87% of the samples, FUM in 73% and DON in 40% of the corn flake samples, at mean levels of 13.6, 255.1 and 87.3 µg/kg, respectively, with one sample (7%) exceeding the maximum permitted levels for both ZEA and DON. Our study showed that the levels of AFB1, OTA, and ZEA in the cornflake samples were below the maximum permissible values. The levels of contamination found among studies may be influenced by differences in analytical methods, manufacturing practices, geographical and climatic conditions, and storage conditions in both countries.

One possible approach in managing mycotoxins in grain chain is the application of a food safety management system (FSMS), which is a system of prevention, preparedness and own-check activities to manage food safety and hygiene in a food business. An FSMS should be seen as a practical tool to control the food production environment and process and to ensure final products are safe for consumption. It includes prerequisite programs (PRPs) [such as GAP (Good Agricultural Practices), GMP (Good Manufacturing Practice), GSP (Good Storage Practices), GHP (Good Hygiene Practices)], HACCP (Hazard Analysis and Critical Control Point)—based procedures, and other management policies and interactive communication in order to ensure traceability and efficient recall systems^21^.

The strengths of our study include the fact that it was the first of its kind in Lebanon and Arab region to assess the safety of packed cornflakes marketed in the country, in terms of five mycotoxins content. In addition, our study included all cornflake SKUs available in the Lebanese market. It is worth to note that most of the SKUs were made in Lebanon since due to the current Lebanese pound exchange rate crisis, imported food products became no longer affordable to the majority of Lebanese citizens, and thus there has been a shift towards purchasing local and more affordable brands. Our analysis was done using ELISA, which is reliable, yet HPLC is the golden analytical method when measuring mycotoxins. Future studies are recommended to use HPLC, in addition to ELISA, for determining mycotoxins in cornflakes. In addition, a food frequency questionnaire study can be carried out to assess the exposure of different age groups to mycotoxins from cornflakes consumption in Lebanon.

## Concluding remarks

In conclusion, our study sheds light on the presence of mycotoxins in cornflakes marketed in Lebanon, which raises important concerns regarding food safety and public health. The detection of mycotoxins, particularly DON, OTA and FUM, in some samples above the EU limits highlights the need for continuous monitoring and control measures to minimize mycotoxin contamination in these products. The higher levels of mycotoxins in locally produced SKUs call for stricter quality control measures in the manufacturing process to ensure consumer safety. With cornflakes being a popular breakfast choice in Lebanon and globally, further investigations should be conducted to assess the potential risks posed to consumers, especially vulnerable populations like children. Our findings emphasize the importance of ongoing research and regulatory efforts to safeguard the quality and safety of cornflake products and promote consumer confidence in the food supply chain. It is crucial for both manufacturers and regulatory authorities to collaborate in implementing effective strategies to mitigate mycotoxin contamination in cornflakes and ensure the provision of safe and nutritious food products for the Lebanese population and beyond.

## Data Availability

All data generated or analysed during this study are included in this published article.
